# The Impact of a Slow-Release Large Neutral Amino Acids Supplement on Treatment Adherence in Adult Patients with Phenylketonuria

**DOI:** 10.3390/nu12072078

**Published:** 2020-07-14

**Authors:** Alessandro P. Burlina, Chiara Cazzorla, Pamela Massa, Christian Loro, Daniela Gueraldi, Alberto B. Burlina

**Affiliations:** 1 Neurological Unit, St. Bassiano Hospital, Via dei Lotti 40, 36061 Bassano del Grappa, Italy; alessandro.burlina@aulss7.veneto.it; 2 Division of Inherited Metabolic Diseases, Department of Women and Children’s Health, University Hospital of Padova, Via Giustiniani 3, 35128 Padova, Italy; chiara.cazzorla@aopd.veneto.it (C.C.); pamela.massa@aopd.veneto.it (P.M.); christian.loro@aopd.veneto.it (C.L.); daniela.gueraldi@aopd.veneto.it (D.G.)

**Keywords:** phenylketonuria, large neutral amino acids, health-related quality of life, adherence, adult patients, PKU-QoL

## Abstract

The gold standard treatment for phenylketonuria (PKU) is a lifelong low-phenylalanine (Phe) diet supplemented with Phe-free protein substitutes. Adherence to therapy becomes difficult after childhood. Supplementing with large neutral amino acids (LNAAs) has been proposed as an alternative medication to Phe-free protein substitutes (i.e., amino acid mixtures). The aim of this study was to evaluate adherence to therapy and quality of life (QoL) in a cohort of sub-optimally controlled adult PKU patients treated with a new LNAA formulation. Twelve patients were enrolled in a 12-month-trial of slow-release LNAAs (1g/kg/day) plus a Phe-restricted diet. Medication adherence was measured with the Morisky Green Levine Medication Adherence Scale; the QoL was measured using the phenylketonuria-quality of life (PKU-QoL) questionnaire. Phe, tyrosine (Tyr) levels, and Phe/Tyr ratios were measured fortnightly. Before treatment, 3/12 patients self-reported a ‘medium’ adherence to medication and 9/12 reported a low adherence; 60% of patients reported a full adherence over the past four weeks. After 12 months of LNAA treatment, all patients self-reported a high adherence to medication, with 96% reporting a full adherence. Phe levels remained unchanged, while Tyr levels increased in most patients. The Phy/Tyr ratio decreased. All patients had a significant improvement in the QoL. LNAAs may give patients a further opportunity to improve medication adherence and, consequently, their QoL.

## 1. Introduction

Phenylketonuria (PKU; OMIM 261600) is a rare genetic disease caused by mutations of the gene encoding for phenylalanine hydroxylase enzyme (PAH; EC1.14.16.1) [1,2]. 

PKU patients are detected by neonatal screening and start a low-phenylalanine (Phe) diet soon after birth [3]. The gold standard therapy for patients with classical PKU is a Phe-restricted diet with the supplementation of Phe-free protein substitutes [4]. The Phe-restricted diet is based on three components: (1) natural protein and Phe restriction according to Phe tolerance, (2) supplementation with protein substitutes (some protein substitutes are not 100% Phe-free, since glycomacropeptide-based protein substitutes are available in several countries), (3) the prescription of special low-protein foods (SLPF) in order to achieve energy requirements for growth and maintenance. The diet has proven efficacy in preventing intellectual disability, but adherence to this therapy is difficult, especially for adolescent and adult patients, and these difficulties could influence many aspects of the patients’ social and emotional lives [5,6]. Difficulty in maintaining blood Phe values within the recommended range (<360 µmol/L and <600 µmol/L according to different ages) [7,8] is related to many issues, including cognitive, emotional, physiological, and cultural factors [9].

According to the published WHO report, adherence rates to long-term therapies in developed countries average only 50% [10]. The poor adherence in PKU patients may manifest in different ways, such as the relaxation of dietary restrictions, failure to take amino acid mixtures (AAMs), not attending regular clinic appointments, and a lack of blood Phe monitoring [11]. Treatment non-adherence has an important consequence on the outcome. The most relevant PKU outcome reported in the literature is a biochemical outcome, that of plasma Phe levels [12]. Medford and colleagues reviewed 29 studies that examined factors related to a poor adherence in children, adolescents, and adult patients with PKU. They recognized that increasing age is significantly related to a progressive reduction in metabolic control [13]. Nevertheless, it has been reported that the proportion of samples with a Phe concentration that is higher than recommended increases with age, while the number of samples sent to the center and the number of outpatient visits decrease significantly [14]. In summary, a full adherence for adult PKU patients cannot be achieved with current treatments.

In another study, we showed that adherence to dietary recommendations among Italian adult PKU patients was poor: Less than half (42%) reported completely following a low-Phe nutritional plan. In respect to the mean daily use of AAMs, in the same study, only 18.5% of patients reported an optimal consumption. The most relevant factors were the negative impact of using AAM powdered products on socializing and difficulties in using it in a working environment because of practical and embarrassing features, as well as unpractical features during traveling. Furthermore, palatability was another negative issue raised by patients [15]. Alternative strategies include the use of large neutral amino acids (LNAAs) [16,17], glycomacropeptide (GMP) [18], and pharmacological treatments such as sapropterin dihydrochloride (BH4) [19] and phenylalanine ammonia lyase enzyme (PEG-PAL) [20].

Large neutral amino acids can compete for transport across the blood–brain barrier (BBB) via a family of transport proteins called the L-system. Among these proteins, the large neutral amino acid transporter designated LAT1 and its glycoprotein subunit called 4F2hc are selective for the BBB transport [16]. The role of LNAAs and their transport into the brain in PKU patients was described in the 1950s by Christensen, who proposed that high blood concentrations of Phe could interfere with the transport of other LNAAs into the brain [21]. Indeed, the modification of amino acid transport across the BBB plays an important role in the pathogenesis of PKU, with unbalancing of Phe and other LNAAs at the transport level, as well as the subsequent disruption of brain amino acid homeostasis [16,22]. Therefore, LNAA supplementation may have several important consequences: the reduction of brain Phe concentrations, the increase of brain neurotransmitter concentrations, and the increase of brain essential amino acid concentrations [23]. 

Recently, in a group of adult patients with PKU and a poor adherence to a restricted diet, we studied the effect of a marked formulation of LNAAs (Neutrafenil Micro R^®^, PIAM Farmaceutici, Italy) characterized by the incorporation of the amino acids in coated microgranules for prolonged release [16]. We showed a significant increase of tyrosine (Tyr) levels and, consequently, an important decrease of the Phe/Tyr ratio in the long-term (after 12 months); Phe levels remained unchanged. To date, however, no studies have been performed to investigate medication adherence in adult PKU patients treated with this LNAA formulation.

The aim of this study was to measure, with a combination of methods (objective, subjective measurement, and biochemical measurements), medication adherence to this new LNAA formulation in a cohort of adult patients with classic PKU.

## 2. Materials and Methods 

### 2.1. Study Population

Fifty-seven patients with classical PKU on diet therapy were enrolled from the Division of Inherited Metabolic Diseases at the University Hospital of Padova, Italy. The diagnosis of PKU was performed by neonatal screening, and all were treated with a Phe-restricted diet from birth.

Twelve out of 57 adult PKU patients (age 19–38 years; mean ± standard deviation (SD) of 29.6 ± 6.8 years) were selected to participate in the study because of their persistent (over a 12-month period) low adherence to the Phe-restricted diet. All 12 patients had a PAH genotype and a tetrahydrobiopterin (BH4) loading test showing a lack of response to the PAH cofactor sapropterin dihydrochloride. All patients of our cohort had normal intelligence quotient (IQ) scores (IQ >70), no history of psychiatric illness, and were able to complete the questionnaires without assistance.

The mean ± SD value of amino acid intake at baseline was: 20.18 ± 9.3 g/day of natural protein and 51.5 ± 7.4 g/day of AAMs. The mean value of protein intake over the 12-month LNAA period was unchanged for natural protein and 45.7 ± 9.8 g/day from the LNAA formulation. All patients reported that, due to their commitments, they generally had lunch or dinner out of the home, with a high intake of Phe (3–4 times the prescribed value).

The body mass index (BMI) for all patients was in the normal range (mean 23.7 ± 3.0; normal range <25), except for one patient with a BMI of 37.2, and was unchanged after 12 months of treatment.

Table 1 shows the demographic characteristics of the enrolled patients.

The LNAA formulation used in the study was Neutrafenil Micro R^®^ (PIAM Farmaceutici S.P.A., Italy). The composition of the product is reported in Supplementary Table S1.

The LNAA formulation consisted of a granulation that could easily be swallowed with water or other liquids. The amino acids were embedded into micro-granules coated with a methylcellulose film to prevent the unpleasant taste related to the amino acid mixture. The container cap was designed so that it can be used to measure 17 grams of amino acids. The supplement, at a dose of 1g/kg body weight, was taken three times daily, at breakfast, lunch, and dinner time, over a 12-month period. The dose of LNAAs was calculated by the dietitian every six months. The LNAA formula completely replaced the previous AAM formula, and no changes were made to their SLPF supplementation. Due to the lack of vitamins and micronutrients in this formula, these supplements were given according to the European PKU guidelines [4]. Patients were asked to continue their regular dietary habits.

Dried blood spots (DBS) were collected at home as part of the routine follow-up for adult PKU patients and sent to our center for the biochemical measurement of Phe and Tyr levels [16]. 

The principles of good clinical practice (GCP) were adhered to throughout the study, in accordance with the Declaration of Helsinki (and its amendments) and the International Conference on Harmonisation (ICH)/GCP guidelines. The study was performed in compliance with local regulatory requirements (EC 4775/AO/19 Provincia di Padova, Azienda Ospedaliera di Padova). Written informed consent was obtained from all participants.

### 2.2. Methods for Therapy Adherence Assessment

At baseline, we assessed subjective treatment adherence with the four-item Morisky Green Levine Medication Adherence Scale (MGLS) [24]. The self-assessed MGLS questionnaire is used in clinical settings to measure therapy adherence for chronic diseases.

The MGLS includes four questions with yes/no response options and results in a score ranging from 0 (high) to 4 (low) to obtain measures of three levels of medication adherence (high, medium, low). The ease of use of this scale allows it to be easily integrated into clinical practice [25].

As a complementary survey to the MGLS, we added three questions about the frequency of non-adherence as a report of subjective perception by the patients. The three questions were: No. 1): the frequency of leftover drug (i.e., AAMs or LNAAs) in the past 4 weeks, as an indicator of non-continuous (less than necessary) therapy (“leftover question”); No. 2): the frequency of insufficient stock of drug (i.e., AAMs or LNAAs) in the past 4 weeks, as an indicator of excess consumption (more than necessary) of therapy (“insufficient stock question”); and No. 3): the frequency of the missed administration of drug (i.e., AAMs or LNAAs) in the past four 4 weeks (“missed administration question”). 

Finally, we also asked patients to indicate a subjective measurement of the percentage of time that they were fully adherent in the past 4 weeks.

### 2.3. Methods for Quality of Life Assessment

The quality of life (QoL) in our cohort of patients was measured with the phenylketonuria-specific phenylketonuria-quality of life (PKU-QoL) questionnaire, an age-specific QoL questionnaire [26,27].

The assessments were repeated, using both instruments, twelve months after the introduction of LNAA therapy.

### 2.4. Statistical Analysis

The data analyzed in this research were category related. The Wilcoxon paired sample *t*-test was adopted to compare the sets of data acquired from the patients before and after the start of the LNAA therapy. Significant values were considered when *p* < 0.05. Categorical variables are expressed as number (%), and quantitative variables are expressed as mean ± SD.

## 3. Results

### 3.1. Biochemical Measurement

In the 12-month period prior to the introduction of LNAAs, the mean ± SD Phe and Tyr values ranged from 628 ± 148 to 1033 ± 198 μmol/L and from 32 ± 7 to 87 ± 35 μmol/L, respectively, while the mean ± SD Phe/Tyr ratio ranged from 9.7 ± 2.2 to 19.9 ± 2.8 μmol/L. The DBS measurement frequency ranged from 5 to 38 (median = 19): three patients (25%) sent more than 24 DBS samples. The mean Phe and Tyr values, Phe/Tyr ratio, and DBS frequency before and after LNAA treatment are shown for each patient in Table 2, and the Phe and Tyr values and Phe/Tyr ratios for the overall patient population are shown in Table 3. Overall, the mean ± SD Phe and Tyr values ranged from 736 ± 93 to 1269 ± 265 μmol/L and from 59 ± 15 to 108 ± 35 μmol/L, respectively, while the mean ± SD Phe/Tyr ratio ranged from 8 ± 1.4 to 20.6 ± 7.6 μmol/L. The frequency of DBS measurement ranged from 14 to 39 (median = 23.5) over the 12-month treatment period: seven patients (58%) sent more than 24 DBS samples. 

For most patients, the mean Phe levels were similar before and during the 12-month treatment period with LNAAs, whereas Tyr levels increased significantly in 11 out of 12 patients (92%) (mean 75 ± 16 μmol/L; *p* = 0.0195). Compared with before the introduction of LNAAs, the mean Phe/Tyr ratio decreased significantly in 10 out of 12 patients (83%) (mean 12 ± 3 μmol/L; *p* < 0.05) after 12-months of treatment with LNAAs; in two patients, the Phe/Tyr ratio showed a small increase (mean 17 ± 5 μmol/L; *p* < 0.16). 

All 12 patients completed the 12-month study with LNAAs. At baseline and at the end of the study, patients had a mean ± SD weight of 70.0 ± 18 and 70.4 ± 18 kg, respectively, as well as a mean ± SD BMI index of 24.80 ± 4.83 and 24.92 ± 5.11, respectively. Neither weight nor BMI were significantly different when compared before to and after the introduction of LNAAs (*p* = 0.57 and *p* = 0.95, respectively). 

### 3.2. Medication Adherence Pre- and Post-LNAA

**Figure 1:**
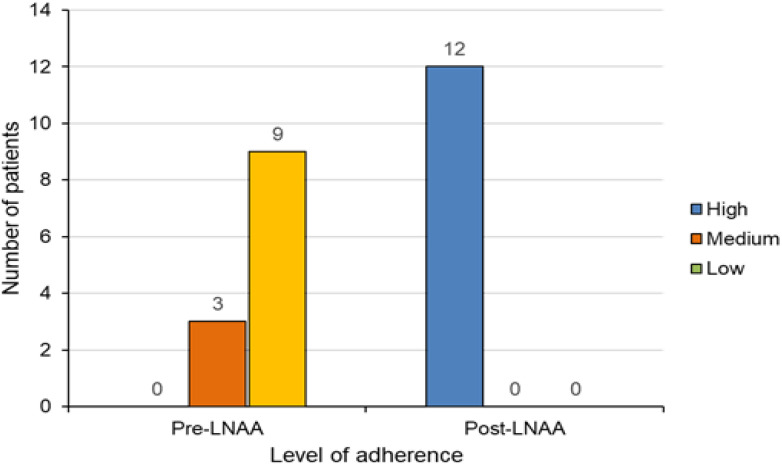
Patients’ subjective assessment of the level of therapy adherence over the past 4 weeks.

During treatment with AAMs, 3/12 patients reported a medium level of medication adherence (average value of 1), and 9 out of 12 reported a low level of medication adherence (average value of 3) (Figure 1). After 12 months with LNAAs only and the suspension of AAMs, all patients reported a high level of medication adherence (average value of 0) (Figure 1). 

Pre-LNAAs, a mean of 52% (range 20–99%) of patients had reported a full adherence over the previous four weeks. This had increased to a mean of 96% (90–100%) adherence over the previous four weeks post-LNAAs. 

**Figure 2:**
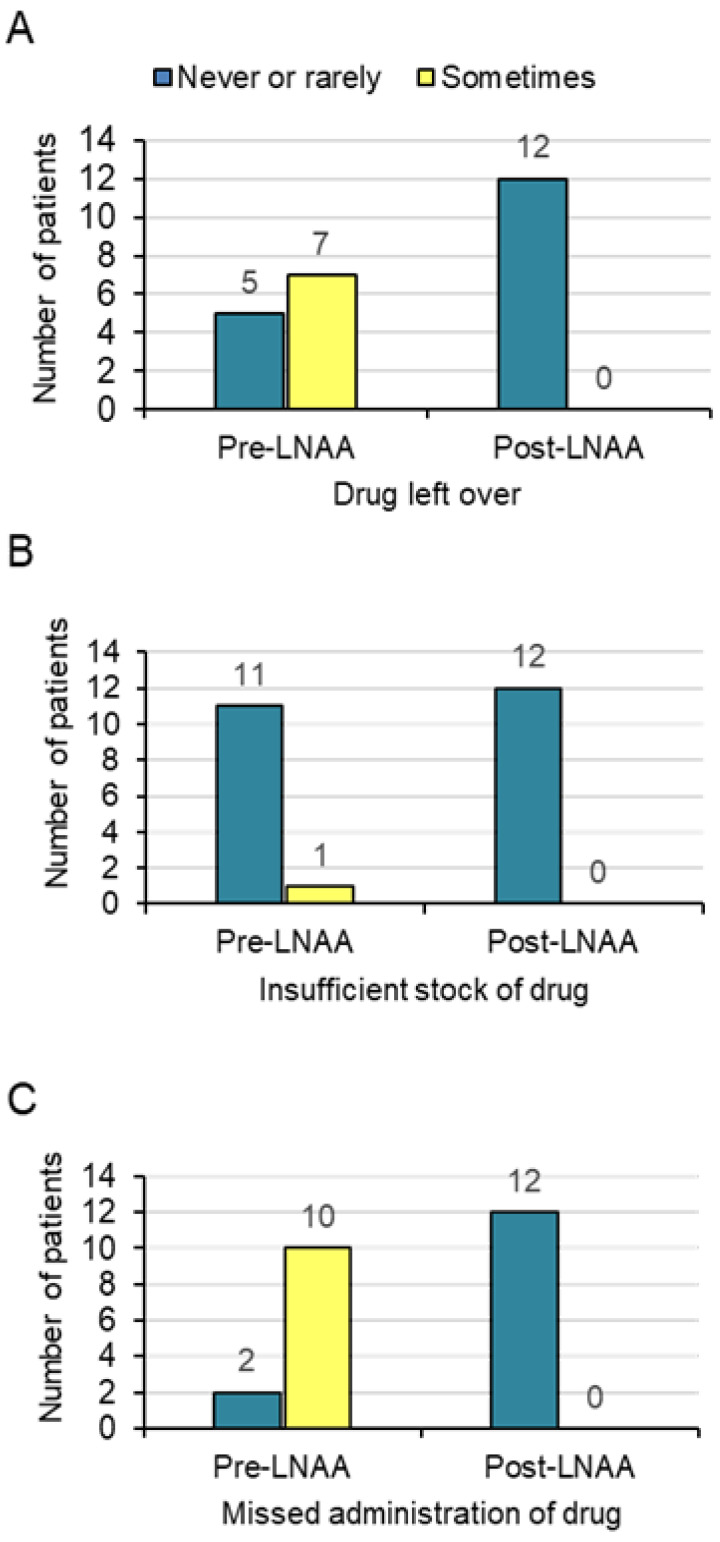
Frequency of non-adherence expressed by 3 questions relating to frequency over the past 4 weeks of (**A**) "Leftover drug," (**B**) "Insufficient stock of drug," and (**C**) "Missed administration of drug."

Considering the three questions assessing the perceptions of the patients on the frequency of non-adherence, in response to the “Leftover question,” pre-LNAAs, 5/12 patients initially reported that they “never or rarely,” 7/12 reported “sometimes,” and no patient reported that they “often” had drugs left over (Figure 2A). Twelve months post-LNAAs, all patients reported that they "never" had drugs left over in the previous four-week period (Figure 2A)

In response to the “Insufficient stock question,” pre-LNAAs, 11/12 patients reported that they “never or rarely” and one reported that they “sometimes” had insufficient stock. No patient reported that they "often" had insufficient stock (Figure 2B). Twelve months post-LNAAs, all patients reported "never" to the question of whether they had insufficient stock in the previous four-week period (Figure 2B).

Pre-LNAAs, in response to the “Missed administration question,” 2/12 patients reported that they “never or rarely” and 10/ 12 reported that they “sometimes” missed the administration of the drug. No patient reported that they “often” missed an administration (Figure 2C). Twelve months post-LNAAs, 9/12 patients reported that they "never" missed drug administration and 3/12 “rarely” missed an administration (Figure 2C).

### 3.3. PKU-QoL Pre- and Post-LNAA Introduction

We compared the variables by considering the answers of patients with a diet and AAMs and the answers of the same patients 12 months later, after AAMs had been replaced with LNAAs. The results showed a significant difference (*p* < 0.05) in the following domains and individual items (Table 4).

“Your health:” There was a statistical difference related to the question about aggressiveness. The patients reported a decrease in the frequency of aggressiveness 12 months after the introduction of LNAAs.

“Your PKU diet and supplements:” Twelve months after the introduction of LNAAs, there was a statistical difference related to four questions: (1) the difficulty of adherence to a diet therapy, (2) the taste of supplements, (3) thinking about eating food that was not part of their diet, and (4) difficulties related to carrying the supplements during business trips or while on holiday. There was a reduced difficulty in following the diet, a perceived improved taste of supplements, fewer thoughts about the regular food not being included in the diet, and fewer difficulties in managing supplements during everyday life were reported by the patients. 

“Your daily life with PKU:” Patients reported a significant trend to find it less inconvenient to carry their supplements when they were going on business trips or on holiday twelve months after the introduction of LNAAs.

“Your feelings in general about PKU:” No statistical difference was found.

## 4. Discussion

The WHO defines adherence to long-term treatment as “the extent to which a person’s behavior, taking medication, following a diet, and/or executing lifestyle changes, corresponds with agreed recommendations from a health care provider” [10].

The term adherence “presumes the patient’s agreement with the recommendations, whereas compliance implies patient passivity” [28]. Therefore, compliance and adherence cannot be used interchangeably. We should consider that compliance can be more easily achieved in a pediatric setting; meanwhile, adherence implies awareness and active collaboration, which can be obtained only with maturity, which comes with adulthood. Indeed, therapeutic adherence from patients with chronic diseases requires the involvement of patients in the therapeutic decision process, especially when the type of therapy influences the patient’s psychological well-being and, consequently, the QoL [29].

The measurement of medication adherence is a complex issue that is influenced by the patient’s behavior. We can distinguish three approaches: (1) objective measurements (e.g., counting the pills, examining pharmacy refill records), (2) subjective measurement (obtained by asking patients, family members, and caregivers), and (3) biochemical measurements obtained by measuring (e.g., in biofluids) a compound present in the medication as a direct marker of the medication taken. Usually, a combination of these measures is implemented to assess patient adherence.

However, to our knowledge, the subjective measurement of medication adherence, which can be tested with a self-reporting questionnaire, has not yet been used for adult PKU patients.

In our study, we investigated the medication adherence and the subsequent QoL of 12 patients with PKU who had shown a poor adherence to the AAMs (no diet changes were implemented).

The LNAA product used in the current study is very rich in Tyr and tryptophan (not measured in our patients) in a new slow-release formulation. The biochemical outcome (Phe levels) of our patients remained unchanged over the duration of our study. Indeed, most of the patients in our cohort showed nonsignificant changes in blood Phe levels at the end of the LNAA treatment period, which was contrary to some previously published studies [30,31,32]. However, previous studies were carried out over a very short period of time (less than 12 months) [33], whereas patients in the current study were treated with LNAAs for a 12-month period. In our study, even in the short term (less than six months), a decrease in Phe levels was detected but Phe levels returned to baseline levels for the majority of patients by the end of the 12-month treatment period.

For our study, the measurement of Tyr levels represented the biochemical measurement of medication adherence. We observed a remarkable increase in blood Tyr levels with a reduction of the Phe/Tyr ratio. These data can be explained by the regular consumption of this extended-release LNAA formulation that is rich in Tyr in comparison with the other previously prescribed AAMs. Moreover, these data emphasize that patients can improve their adherence to a medical food (for example, the LNAA medication in our study), but they do not imply that PKU patients will also strictly follow their diet regime, including SLPF.

As a measurement of objective adherence, we counted the number of DBS sent from the patients. After 12 months of treatment with LNAAs, we were able to detect an increase of DBS for all patients except one; three patients doubled the number of DBS sent to our center (Table 2).

Our study also showed that the subjective measurement of adherence is feasible for adult PKU patients. Regarding therapeutic adherence, after 12 months of LNAA consumption, the patients perceived a significant improvement in therapy adherence, as reported by the more regular consumption of the prescribed medication.

In fact, the analysis of the MGLS data related to the medication adherence highlighted that patients perceived a medium-low level of adherence before the introduction of LNAAs. With the introduction of LNAAs, we observed an increase in the level of the subjective perception of adherence. In fact, all patients of our cohort reported that the level of their subjective adherence became “high”, while the subjective frequency of non-adherence became “never”. Regarding non-adherence, the analysis of patients’ responses showed this was due to forgetfulness, running out of stock (patients forgot it to refill it in time), feelings of well-being, or other reasons not specified.

Even though we detected an improvement in medication adherence, we are not sure that diet adherence was ameliorated. Regarding the question “Your PKU diet and supplements” the patients reported a tendency to have fewer difficulties in following the diet, but blood Phe levels remained elevated, as reported in our previous study [16].

In our study, we also analyzed the QoL, because we believed that better adherence could also impact on the QoL, as previously reported [34,35]. Indeed, the patients who had replaced their AAMs with LNAAs reported fewer episodes of aggressiveness and irritability (fewer arguments and quarrels within their families/partners). We also noticed that the patients were complaining less about palatability (this LNAA formulation is tasteless), and, ultimately, this represented a further stimulus to follow the therapy. In addition, the patients who had switched to LNAAs reported fewer “recurring thoughts” about a free diet without any restrictions.

Our study confirmed that clinicians, psychologists, and dieticians who are taking care of adult PKU patients should be aware of worsening therapy adherence during aging (i.e., from adolescence to young adulthood). Moreover, they should provide additional support and develop coping strategies to face this crucial issue for an appropriate long-term follow-up.

Our study had two limitations. The first concerned the short period of follow-up, which was 12 months in our case. Indeed, even though this was one of the longest periods for LNAA treatment reported in the literature, we believe it was still not enough for a chronic condition like PKU. Data from the literature have shown that a good biochemical outcome (Phe levels) for adult PKU patients can be obtained from adult PKU patients in a time interval of one-to-six months [31,32]. We could define this period of time as a sort of “honeymoon”, characterized by a patients’ strong motivation, which is a major empowerment for any therapy adherence.

The second limitation was related to the self-rating instruments, which could be influenced by some bias, such as memory bias, patient education, the complex psychological phenotype of adult PKU patients, and more practical issues, such as the availability of web-based material [36,37]. 

Nevertheless, we believe that adherence to long-term therapy should be investigated for adult patients affected with inherited metabolic diseases. Among these diseases, PKU is certainly worthy of consideration because it is the most common inherited metabolic disease, and its life-long span requires the appropriate management of available therapy.

## 5. Conclusions

Our study showed that medication adherence should be carefully assessed in adult PKU patients and should be a priority objective for any adult PKU patient. Any dedicated PKU team should apply appropriate instruments of empowerment to motivate the adult PKU patient to understand that better diet adherence means a better outcome. 

## Figures and Tables

**Table 1:** Demographic characteristics of the cohort of adult phenylketonuria (PKU) patients (n = 12).

Patients	Gender	Age, Years	Molecular Analysis	Marital Status	Educational Level ^1^	Professional Status	Leisure Time
**1**	M	38	c.473G>A/ c.1315+1G>A	Unmarried	8	Agriculture	Not specified
**2**	M	33	c.473G>A/ c.526C>T	Married	18	Insurance agent	Sport
**3**	M	30	c.473G>A/ c.473G>A	Married	18	Unemployed	Intellectual
**4**	M	35	c.842+3G>C in heterozygosis	Married	13	Office worker	Not specified
**5**	M	26	c.47_48delCT/ c.1315+2T>C	Unmarried	18	Freelance	Intellectual
**6**	F	32	c.842C>T/ c.1315+1G>A	Married	18	Teacher	Social activity
**7**	F	28	c.842C>T/ c.1315+1G>A	Unmarried	13	Office worker	Not specify
**8**	F	22	c.842C>T/ c.1315+1G>A	Unmarried	13	Unemployed	Social activity
**9**	M	32	c.1222C>T/ c.1315+1G>A	Unmarried	13	Sales	Not specify
**10**	M	40	c.782G>A/ c.782G>A	Married	13	Freelance	Sport
**11**	F	20	c.782G>A/ c.1066-11G>A	Unmarried	13	Unemployed	Not specify
**12**	F	19	c.1222C>T/ macro deletion in exon 3	Married	13	Unemployed	Sport

M = male; F = female; PKU = phenylketonuria. ^1^ expressed in years.

**Table 2:** Phenylalanine (Phe), tyrosine (Tyr), and Phe/Tyr ratio and frequency of dried blood spots (DBS) of each patient, before and after the introduction of large neutral amino acid (LNAA) treatment.

Patient	12 Months before LNAA				12 Months after LNAA			
	Phe Values	Tyr Values	Phe/Tyr Values	DBS Frequency	Phe Values	Tyr Values	Phe/Tyr Values	DBS Frequency
**1**	790 ± 80	47 ± 8	17.2 ± 2.5	8	825 ± 114	68 ± 10	12.4 ± 2.4	17
**2**	1033 ± 198	54 ± 10	19.5 ± 4.4	5	1269 ± 265	65 ± 14	20.6 ± 7.6	14
**3**	983 ± 142	32 ± 7	31.5 ± 7.3	10	907 ± 166	60 ± 8	15.4 ± 3	20
**4**	790 ± 118	83 ± 14	9.7 ± 2.2	18	769 ± 144	98 ± 18	8 ± 1.4	34
**5**	838 ± 179	57 ± 12	15.7 ± 6.6	20	889 ± 149	79 ± 19	11.7 ± 2.7	24
**6**	880 ± 160	49 ± 11	18.3 ± 4	38	975 ± 148	68 ± 29	16 ± 5	39
**7**	779 ± 108	87 ± 35	10.6 ± 5	25	736 ± 93	86 ± 41	10.5 ± 4.9	27
**8**	628 ± 148	50 ± 13	13 ± 5.2	26	790 ± 147	59 ± 15	14 ± 4.5	29
**9**	808 ± 135	59 ± 7	19.9 ± 2.8	12	925 ± 139	79 ± 13	11.9 ± 1.8	18
**10**	842 ± 94	75 ± 12	11.5 ± 2.3	23	879 ± 91	108 ± 35	8.5 ± 1.9	36
**11**	765 ± 234	58 ± 11	13.4 ± 3.9	23	760 ± 124	60 ± 11	13 ± 2.7	24
**12**	823 ± 117	53 ± 19	16.9 ± 5	18	1000 ± 163	68 ± 17	15.2 ± 3.7	15

Data for Phe (phenylalanine), Tyr (tyrosine), and Phe/Tyr are mean ± SD μmol/L. DBS = dried blood spot; LNAA = large neutral amino acid; SD = standard deviation. DBS were sent from home. The frequency requested by the center was twice a month.

**Table 3:** Comparison between the mean values of Phe, Tyr, and Phe/Tyr ratio of all patients in the twelve months before starting LNAA treatment and during LNAA treatment.

Overall Patient Population	Prior to LNAA Treatment	During LNAA Treatment	*p* Value
Phe	752 ± 143	894 ± 145	0.0522
Tyr	59 ± 13	75 ± 16	0.0195
Phe/Tyr	16± 4	12 ± 3	0.049

Data for Phe (phenylalanine), Tyr (tyrosine), and Phe/Tyr are mean ± standard deviation (SD) μmol/L. LNAA = large neutral amino acid.

**Table 4:** Phenylketonuria-quality of life (PKU-QoL) score: data on specific dimensions and items after LNAA treatment.

Dimension	Item	*p* Value
Your Health	In the past 7 days, I became aggressive	0.044
Your PKU diet and supplements	In the past 7 days, it was hard to take my supplements several times a day	0.042
	In the past 7 days, it was hard to only eat/drink what I should	0.047
	In the past 7 days, my supplements tasted: very good/good/ok, bad, very bad/I don’t take supplements	0.017
	In the past 7 days, I thought about eating food that was not on my diet	0.047
Your daily life with PKU	Currently, it is inconvenient to carry my supplements with me when I am going on business trips or on holiday	0.040

PKU-QoL = phenylketonuria-quality of life questionnaire; LNAA = large neutral amino acid.

## Supplementary Materials

The following are available online at https://www.mdpi.com/2072-6643/12/7/2078/s1, Table S1: Nutritional composition per 100 g of the large neutral amino acids formulation (Neutrafenil Micro R^®^).

## References

[1] Blau N., Van Spronsen F.J., Levy H.L. (2010). Phenylketonuria. Lancet.

[2] Blau N. (2016). Genetics of Phenylketonuria: Then and Now. Hum. Mutat..

[3] Camp K.M., Parisi M.A., Acosta P.B., Berry G.T., Bilder D.A., Blau N., Bodamer O.A., Brosco J.P., Brown C.S., Burlina A. (2014). Phenylketonuria Scientific Review Conference: State of the science and future research needs. Mol. Genet. Metab..

[4] Van Wegberg A.M.J., Macdonald A., Ahring K., Belanger-Quintana A., Blau N., Bosch A.M., Burlina A., Campistol J., Feillet F., Gizewska M. (2017). The complete European guidelines on phenylketonuria: Diagnosis and treatment. Orphanet J. Rare Dis..

[5] Macdonald A., Gokmen-Ozel H., Van Rijn M., Burgard P. (2010). The reality of dietary compliance in the management of phenylketonuria. J. Inherit. Metab. Dis..

[6] Enns G., Koch R., Brumm V., Blakely E., Suter R., Jurecki E. (2010). Suboptimal outcomes in patients with PKU treated early with diet alone: Revisiting the evidence. Mol. Genet. Metab..

[7] Van Spronsen F.J., Van Wegberg A.M., Ahring K., Belanger-Quintana A., Blau N., Bosch A.M., Burlina A., Campistol J., Feillet F., Gizewska M. (2017). Key European guidelines for the diagnosis and management of patients with phenylketonuria. Lancet Diabetes Endocrinol..

[8] Vockley J., Andersson H.C., Antshel K.M., Braverman N.E., Burton B.K., Frazier D.M., Mitchell J., Smith W.E., Thompson B.H., Berry S.A. (2014). Phenylalanine hydroxylase deficiency: Diagnosis and management guideline. Genet. Med..

[9] Levy H., Lamppu D., Anastosoaie V., Baker J.L., DiBona K., Hawthorne S., Lindenberger J., Kinch D., Seymour A., McIlduff M. (2020). 5-year retrospective analysis of patients with phenylketonuria (PKU) and hyperphenylalaninemia treated at two specialized clinics. Mol. Genet. Metab..

[10] Sabaté E., Sabaté E. (2003). Adherence to Long-Term Therapies: Evidence for Action.

[11] Jurecki E., Cederbaum S., Kopesky J., Perry K., Rohr F., Sanchez-Valle A., Viau K., Sheinin M., Cohen-Pfeffer J. (2017). Adherence to clinic recommendations among patients with phenylketonuria in the United States. Mol. Genet. Metab..

[12] Pugliese M., Tingley K., Chow A., Pallone N., Smith M., Rahman A., Chakraborty P., Geraghty M.T., Irwin J., Tessier L. (2020). Outcomes in pediatric studies of medium-chain acyl-coA dehydrogenase (MCAD) deficiency and phenylketonuria (PKU): A review. Orphanet J. Rare Dis..

[13] Medford E., Hare D.J., Wittkowski A. (2017). Demographic and Psychosocial Influences on Treatment Adherence for Children and Adolescents with PKU: A Systematic Review. JIMD Reports.

[14] Macdonald A., Van Rijn M., Feillet F., Lund A., Bernstein L., Bosch A.M., Gizewska M., Van Spronsen F.J. (2012). Adherence Issues in Inherited Metabolic Disorders Treated by Low Natural Protein Diets. Ann. Nutr. Metab..

[15] Cazzorla C., Bensi G., Biasucci G., Leuzzi V., Manti F., Musumeci A., Papadia F., Stoppioni V., Tummolo A., Vendemiale M. (2018). Living with phenylketonuria in adulthood: The PKU ATTITUDE study. Mol. Genet. Metab. Rep..

[16] Burlina A.P., Cazzorla C., Massa P., Polo G., Loro C., Gueraldi D., Burlina A.P. (2019). Large Neutral Amino Acid Therapy Increases Tyrosine Levels in Adult Patients with Phenylketonuria: A Long-Term Study. Nutrients.

[17] Scala I., Riccio M.P., Marino M., Bravaccio C., Parenti G., Strisciuglio P. (2020). Large Neutral Amino Acids (LNAAs) Supplementation Improves Neuropsychological Performances in Adult Patients with Phenylketonuria. Nutrients.

[18] Pena M.J., Pinto A., Daly A., Macdonald A., Azevedo L.F., Rocha J.C., Borges N. (2018). The Use of Glycomacropeptide in Patients with Phenylketonuria: A Systematic Review and Meta-Analysis. Nutrients.

[19] Burlina A., Blau N. (2008). Effect of BH_4_ supplementation on phenylalanine tolerance. J. Inherit. Metab. Dis..

[20] Longo N., Dimmock D., Levy H., Viau K., Bausell H., Bilder D.A., Burton B., Gross C., Northrup H., Rohr F. (2018). Evidence- and consensus-based recommendations for the use of pegvaliase in adults with phenylketonuria. Genet. Med..

[21] Christensen H.N. (1953). Metabolism of Amino Acids and Proteins. Annu. Rev. Biochem..

[22] Van Vliet D., Bruinenberg V.M., Mazzola P.N., Van Faassen M.H.J.R., De Blaauw P., Kema I.P., Heiner-Fokkema M.R., Van Anholt R.D., Van Der Zee Y.A., Van Spronsen F.J. (2015). Large Neutral Amino Acid Supplementation Exerts Its Effect through Three Synergistic Mechanisms: Proof of Principle in Phenylketonuria Mice. PLoS ONE.

[23] Van Spronsen F.J., De Groot M.J., Hoeksma M., Reijngoud D.-J., Van Rijn M. (2010). Large neutral amino acids in the treatment of PKU: From theory to practice. J. Inherit. Metab. Dis..

[24] Morisky D.E., Green L.W., Levine D.M. (1986). Concurrent and Predictive Validity of a Self-reported Measure of Medication Adherence. Med. Care.

[25] Beyhaghi H., Reeve B.B., Rodgers J.E., Stearns S.C. (2016). Psychometric Properties of the Four-Item Morisky Green Levine Medication Adherence Scale among Atherosclerosis Risk in Communities (ARIC) Study Participants. Value Health.

[26] Bosch A.M., Burlina A., Cunningham A., Bettiol E., Moreau-Stucker F., Koledova E., Benmedjahed K., Regnault A. (2015). Assessment of the impact of phenylketonuria and its treatment on quality of life of patients and parents from seven European countries. Orphanet J. Rare Dis..

[27] Regnault A., Burlina A., Cunningham A., Bettiol E., Moreau-Stucker F., Benmedjahed K., Bosch A.M. (2015). Development and psychometric validation of measures to assess the impact of phenylketonuria and its dietary treatment on patients’ and parents’ quality of life: The phenylketonuria—Quality of life (PKU-QOL) questionnaires. Orphanet J. Rare Dis..

[28] Brown M.T., Bussell J.K. (2011). Medication adherence: WHO cares?. Mayo Clin. Proc..

[29] Cuevas C.D.L. (2011). Towards a clarification of terminology in medicine taking behavior: Compliance, adherence and concordance are related although different terms with different uses. Curr. Clin. Pharmacol..

[30] Schindeler S., Ghosh-Jerath S., Thompson S., Rocca A., Joy P., Kemp A., Rae C.D., Green K., Wilcken B., Christodoulou J. (2007). The effects of large neutral amino acid supplements in PKU: An MRS and neuropsychological study. Mol. Genet. Metab..

[31] Matalon R., Michals-Matalon K., Bhatia G., Grechanina E., Novikov P., McDonald J.D., Grady J., Tyring S.K., Guttler F. (2006). Large neutral amino acids in the treatment of phenylketonuria (PKU). J. Inherit. Metab. Dis..

[32] Matalon R., Michals-Matalon K., Bhatia G., Burlina A.B., Burlina A.P., Braga C., Fiori L., Giovannini M., Grechanina E., Novikov P. (2007). Double blind placebo control trial of large neutral amino acids in treatment of PKU: Effect on blood phenylalanine. J. Inherit. Metab. Dis..

[33] Concolino D., Mascaro I., Moricca M.T., Bonapace G., Matalon K., Trapasso J., Radhakrishnan G., Ferrara C., Matalon R., Strisciuglio P. (2016). Long-term treatment of phenylketonuria with a new medical food containing large neutral amino acids. Eur. J. Clin. Nutr..

[34] García M.I., Araya G., Coo S., Waisbren S.E., De La Parra A. (2017). Treatment adherence during childhood in individuals with phenylketonuria: Early signs of treatment discontinuation. Mol. Genet. Metab. Rep..

[35] Waisbren S.E., Rokni H., Bailey I., Rohr F., Brown T., Warner-Rogers J. (1997). Social factors and the meaning of food in adherence to medical diets: Results of a maternal phenylketonuria summer camp. J. Inherit. Metab. Dis..

[36] Stirratt M., Dunbar-Jacob J., Crane H.M., Simoni J.M., Czajkowski S., Hilliard M., Aikens J.E., Hunter C.M., Velligan D.I., Huntley K. (2015). Self-report measures of medication adherence behavior: Recommendations on optimal use. Transl. Behav. Med..

[37] Burlina A.P., Lachmann R.H., Manara R., Cazzorla C., Celato A., Van Spronsen F.J., Burlina A. (2019). The neurological and psychological phenotype of adult patients with early-treated phenylketonuria: A systematic review. J. Inherit. Metab. Dis..

