# Supplementary material for: The Impact of a Slow-Release Large Neutral Amino Acids Supplement on Treatment Adherence in Adult Patients with Phenylketonuria
Source: Nutrients. 2020 Jul 14;12(7):2078. doi: 10.3390/nu12072078 (PMC7400920; doi:10.3390/nu12072078)
Supplement: Supplementary file 1 [file nutrients-12-02078-s001.pdf]

Supplementary Material

The impact of large neutral amino acids on treatment adherence in adult patients with phenylketonuria

Alessandro P. Burlina[1], Chiara Cazzorla[2], Pamela Massa[2], Christian Loro[2], Daniela Gueraldi[2] and Alberto B. Burlina[2],*

[1] Neurological Unit, St. Bassiano Hospital, Via dei Lotti 40, 36061 Bassano del Grappa, Italy; alessandro.burlina@aulss7.veneto.it (A.P.B.)

[2] Division of Inherited Metabolic Diseases, Department of Women and Children's Health, University Hospital of Padova, Via Giustiniani 3, 35128, Padova, Italy; alessandro.burlina@aulss7.veneto.it (APB); chiara.cazzorla@aopd.veneto.it (C.C.); pamela.massa@aopd.veneto.it (P.M.); christian.loro@aopd.veneto.it (C.L); daniela.gueraldi@aopd.veneto.it (D.G.); alberto.burlina@unipd.it (A.B.B)

* Correspondence: alberto.burlina@unipd.it (A.B.B.); Tel.: +39-049 8213569

Supplementary Table S1. Nutritional composition per 100 g of the large neutral amino acids formulation (Neutrafenil Micro R®)

Energy	1686 Kj<br>399 Kcal
Fat	5.3 g
of which saturated fatty acids	5.3 g
Carbohydrates	12.0 g
of which sugar	0
Fiber	5.8 g
Equivalent Protein	70.73 g
Salt	1.6 g
L-arginine	1.92 g
Apartate	4.95 g
L-phenylalanine	0 g
L-isoleucine	10.0 g
L-histidine	3.36 g
L-leucine	12.0 g
L-lysine	5.44 g
L-methionine	2.72 g
L-tyrosine	24 g
L-threonine	2.56 g
L-tryptophan	8.0 g
L-valine	10.0 g
